# Leaf Analyzer: A fully automated and open-source tool for high-throughput leaf trait measurement

**DOI:** 10.1016/j.plaphe.2025.100145

**Published:** 2025-12-26

**Authors:** Tao Hu, Richard Poire, Danielle Way

**Affiliations:** a Research School of Biology, Australian National University, 134 Linnaeus Way, Acton, ACT, 2601, Australia; b Australian Plant Phenomics Network, 134 Linnaeus Way, Acton, ACT, 2601, Australia

**Keywords:** Leaf area, Leaf dimensions, Leaf traits, Leaf damage assessment, Percent herbivory, Plant phenotyping, Leaf segmentation, Unsupervised learning

## Abstract

Accurate and efficient leaf trait measurement is essential for plant phenotyping, agronomy, and ecological studies. In this work, we introduce Leaf Analyzer, a novel open-source, fully automated computer vision-based tool for high-throughput leaf morphological trait measurement such as leaf area, dimensions, perimeter, count, and percent damage. Unlike existing methods that rely on strong foreground-background contrast or controlled imaging conditions, Leaf Analyzer employs an unsupervised clustering approach based on the K-means++ clustering algorithm and a novel Leaf Background Separation (LBS) feature, which combines the L∗ and b∗ channels from CIEL∗a∗b∗ color space and the saturation channel from HSV color space. The proposed method and the LBS feature can effectively distinguish leaves from the background across varying lighting conditions, leaf colors, and camera orientations. To evaluate the performance of the new software, we conducted comprehensive quantitative and qualitative comparison experiments with two widely used software tools — Petiole Pro and LeafByte, demonstrating that Leaf Analyzer achieves superior accuracy and consistency, particularly under challenging imaging conditions. Additionally, we explore methods to further enhance measurement precision, including leaf flattening and the integration of supplementary leaf features such as texture features and color specific features. Beyond leaf trait measurement, we showcase the versatility of Leaf Analyzer in a range of applications, including nondestructive plant phenotyping, seed counting, root trait analysis, leaf area measurement for petri dish-grown plants, plant projected silhouette area or crown projection area estimation, leaf damage assessment, and broader plant science applications, making it a valuable tool for researchers working in laboratory and field environments.

## 1 Introduction

The accurate measurement of leaf area is crucial across a wide range of research fields, as it plays a pivotal role in understanding plant physiology, growth, and productivity. Leaf area strongly influences light interception, which in turn drives photosynthesis [1,2] and transpiration. It also serves as a key variable in numerous models for simulating carbon and water cycles [3], making it an essential parameter in plant science, ecology, and agricultural research.

Historically, researchers have relied on direct methods such as grid counting and planimeters to measure leaf area. While these techniques can yield accurate results, they are time-consuming, labor-intensive, and prone to manual tracing errors, particularly when dealing with large datasets. Advances in sensing technology introduced electronic leaf area meters, such as those developed by Delta-T Devices (Cambridge, UK) and LI-COR (Lincoln, NE, USA). These devices automate measurements but come with drawbacks, including high cost, complex maintenance requirements, and sensitivity to environmental factors such as dirt on belts, obstructed mirrors or lenses, and calibration drift [4]. Additionally, concerns about measurement precision have been raised in various studies [5,6].

Some indirect methods have been proposed to estimate leaf area. These approaches often involve building regression models that correlate leaf size and shape with area. For example, the Montgomery equation [7] and the Gielis equation [8,9] are often used for measuring leaf area. These models are usually species specific, for example the linear regression model for cucumber leaf [10], and the linear mixed-effect model for broadleaf species [11]. Gravimetric methods offer another alternative by estimating leaf area based on weight. For instance, researchers have proposed weighing the projected area of leaves on graph paper against the weight of a known reference area [12] or weighing the entire leaf against a standard leaf section [13]. Similarly, relationships between leaf area and dry or fresh mass have been modelled [14]. Volumetric methods have also been explored. Haghshenas et al. propose to calculate volumetric leaf area by measuring the leaf volumetry divided by leaf thickness [15]. However, these indirect methods are susceptible to manual measurement errors (e.g., errors in measuring leaf dimensions, thickness, or weight) and calibration biases. For instance, the linear mixed-effect model in Ref. [11] achieved a mean absolute error percentage (MAE%) as high as 6.9 %, with an error range of 4.1–13.0 %. Additionally, Rahimikhoob et al. [16] compared five estimation methods and found simple linear regression to be the least accurate.

Advances in image processing have enabled more cost-effective, efficient, and scalable methods for leaf area measurement. These methods leverage algorithms to segment leaves from their backgrounds, calculate leaf area in pixels, and convert it to metric units (e.g., square millimeters). The major challenge in such methods is leaf segmentation. Many approaches rely on manual [17,18] or adaptive thresholding techniques, such as Otsu's method [16,[19], [20], [21], [22]], which require high contrast between the leaf and background. These methods often fail under suboptimal lighting conditions or when shadows are present, limiting their applicability in outdoor environments. To overcome these limitations, researchers have developed supervised classification techniques that train models on leaf pixel samples to distinguish leaves from the background based on color features [23,24]. More recently, deep learning approaches have emerged, offering robust segmentation by extracting reliable features that go beyond simple color cues [6,21,[25], [26], [27]]. However, these supervised learning methods require extensive training datasets, which are typically species-specific, and their generalizability to other species remains limited. Additionally, creating and annotating large datasets for training is time-intensive. While this burden can be alleviated to some extent by semi-supervised or self-supervised methods [[28], [29], [30]], these approaches still require a certain amount of labeled data to achieve reasonable accuracy. They are often species specific as well and can suffer from reduced performance due to their sensitivity to pseudo-label noise.

Another critical component of image processing-based methods is the pixel to mm conversion or scale calibration. Most approaches use a reference object of known size, such as a ruler [[31], [32], [33]], a red/black block [6,16,34], a rectangle [17,35], a checkerboard [18], or a standard paper card [36]. However, reference objects can interfere with leaf segmentation if their color or intensity closely resembles that of the leaves. Moreover, these methods often neglect image distortions caused by skewed camera viewing angle, which can compromise accuracy. To address this issue, geometric correction methods using calibration patterns have been developed. For example, LeafByte [37] employs four black dots for scale calibration and geometric correction, though the detection of the dots can be hampered by poor illumination or interference from background objects. Petiole Pro [38], a commercial software tool, employs a more robust calibration pattern with eight ArUco markers [39] arranged in a circle. While effective, the fixed pattern size of Petiole Pro reduces accuracy when measuring large leaves and limits flexibility.

Our research aims to address these limitations by developing Leaf Analyzer — an open-source, reliable, user-friendly, fully-automatic, and high-throughput software tool for estimating common leaf morphological traits with high accuracy. The tool is designed to be generalizable across species, functional both in laboratory and field environments, and capable of handling challenging imaging conditions such as shadows or varying lighting. Many existing software tools or methods are not fully automatic [31,36,40], including commercial software such as Digimizer, WinFolia, and Petiole Pro. For example, Petiole Pro requires the user to click on each individual leaf in the image to display the measurements to avoid false leaf detections. Unlike these tools, Leaf Analyzer is designed to be as automated as possible. Furthermore, many tools do not account for image distortions [33,34], which emphasizes the need for users to acquire precise and clear images for good accuracy.

Leaf Analyzer incorporates the following key features:

1. Automatic Scale Calibration and Geometric Correction: Leaf Analyzer does not require costly devices or special setups, just a custom-designed pattern featuring four AprilTags [41] on a white background for robust scale calibration and geometric correction, enabling accurate measurements even under challenging imaging conditions. The pattern can be easily printed with a standard office printer.
2. Automatic Leaf Segmentation: The software employs an unsupervised learning method based on K-means++ and a new feature called Leaf Background Separation (LBS), which combines the L∗ and b∗ channels from the CIEL∗a∗b∗ color space with the S channel from the HSV color space. This approach eliminates the need for training datasets and provides robust segmentation across leaves of all shapes and colors (except for white leaves) automatically even under adverse lighting conditions.
3. High-Throughput Processing: Leaf Analyzer supports batch processing, a critical requirement for modern plant phenotyping studies. If images are taken with the same-sized pattern in the background, they can be placed in one folder and batch processed by Leaf Analyzer, with the measurements for each image saved in a single spreadsheet file.
4. Comprehensive Trait Analysis: In addition to measuring leaf area, the software computes other key traits, including leaf count, dimensions, perimeter, percent herbivory, and disease rates.
5. Indoor and Outdoor Compatibility: Leaf Analyzer can be used in both controlled indoor environments and outdoor field conditions, making it suitable for a wide range of research applications.
6. Support for Destructive and Non-Destructive Measurements: The software allows for both destructive sampling (where leaves are removed for analysis) and non-destructive measurements (where leaves remain intact), providing flexibility for different experimental setups.
7. Cross-Platform Availability: Leaf Analyzer is available for multiple operating systems, including Windows, Linux, and macOS, ensuring accessibility across different computing environments.

Owing to the above features, Leaf Analyzer has already been used in peer-reviewed research. For example in recent studies on crops grown in simulated Martian regolith [42,43], Leaf Analyzer was used to measure microgreen leaf areas and their standard deviations.

In comparative evaluations with popular tools such as Petiole Pro and LeafByte, Leaf Analyzer demonstrated significantly higher accuracy, achieving around three times the accuracy (measured using MAE%) of Petiole Pro and 1.7 times that of LeafByte in a simulation test of objects of known area. Additionally, Leaf Analyzer successfully processed images taken in outdoor conditions where the other tools failed, making it particularly suitable for field applications. For further illustration, we provide two video demonstrations (on Youtube: Demo 1, Demo 2 or on Youku: Demo 1, Demo 2) showcasing the software's capabilities in calculating morphological traits and assessing leaf damage.

In the following sections, we present the methodology and implementation of Leaf Analyzer, as well as a comprehensive evaluation of its performance against existing tools.

## 2 Materials and methods

**Fig. 1:**
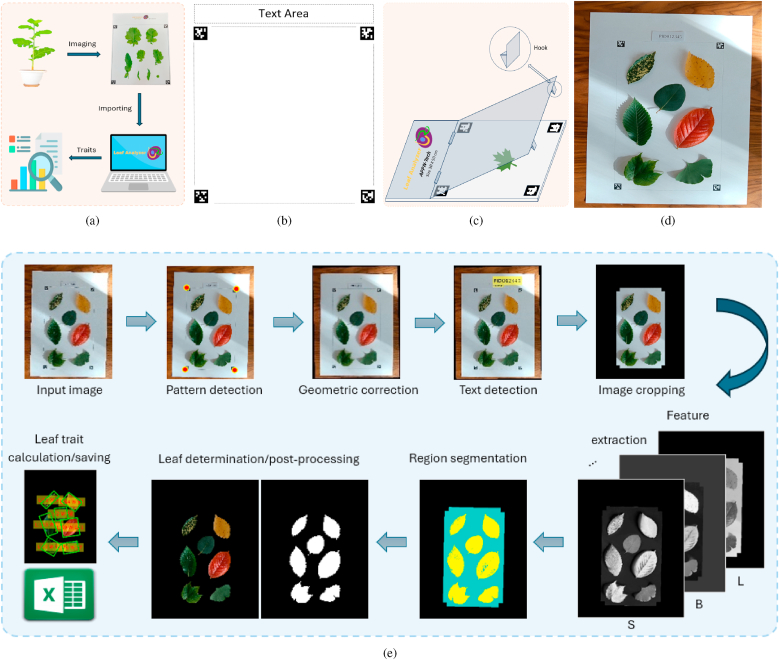
The proposed computer vision-based solution for leaf trait measurement. (a) Overview of the solution, (b) Pattern design, (c) Pattern board with a plastic cover for repeated use and improved accuracy, (d) A sample image, (e) Workflow of the leaf trait calculation process.

Fig. 1a presents our computer vision-based leaf phenotyping solution. To compute leaf traits, we capture images of leaves placed on a background embedded with our custom-designed pattern (see Sec. 2.2 for details). These images are then processed using the developed software tool, Leaf Analyzer, which automatically extracts the desired leaf traits and exports the results into a spreadsheet format (Microsoft Excel or CSV). Fig. 1e outlines the detailed image processing workflow. The following sections describe the key technical aspects of our approach.

### 2.1 Image acquisition

Leaf Analyzer does not require a special imaging device or homogenous lighting for image acquisition. The user can capture images using a standard mobile phone camera in either laboratory or field environments. The only requirement is that the leaves must be placed on a background with the Leaf Analyzer pattern (Sec. 2.2). Unlike many existing methods that require the camera to be strictly perpendicular to the leaf plane (e.g., Refs. [17,33]), Leaf Analyzer can correct for geometric distortions using the background pattern (Sec. 2.2). However, for improved accuracy, it is still recommended to capture images at an appropriate angle and under good lighting conditions. Fig. 1d shows a sample image taken with an OPPO A60 smartphone (an average phone in the market) and a piece of paper printed with our pattern in the background under non-uniform lighting conditions, in which there are leaves of assorted shapes and colors. We use this image as a sample image because in a real phenotyping application, leaves can have variegated colors even if they are from the same species.

### 2.2 Pattern design, image undistortion and scale calculation

Manual calculation of scale using a ruler or a reference object in the image is labor-intensive and prone to errors. This process can be automated by using a pattern made of fiducial markers, such as ARTag, AprilTag, ArUco, and STag. Each marker system has its own advantages and limitations depending on the application and environment. In a comparative study by Ref. [44], the authors evaluated four commonly used fiducial markers and concluded that AprilTag offers superior overall performance in terms of measurement accuracy and detection rate across various conditions. For our application, we select 4 AprilTags from the “tag36h11″ family, which can be easily detected even in complex field environments. The 4 AprilTags are positioned at the four corners of a rectangular region where the leaves are placed. Once the AprilTags are detected, the image undergoes geometric correction via homography transformation. The scale is then computed as the ratio between the rectangle's pixel dimensions and its actual physical dimensions, which are provided by the user.

The pattern reserves a special area on the top for text (Fig. 1b), allowing users to annotate metadata such as plant ID and treatment information. This text can either be handwritten or printed on a label placed within the designated area, as shown in Fig. 1d. The text can be recognized by Leaf Analyzer and saved together with the calculated traits. We restrict the text to a specific area because text detection is computationally intensive. The size of the pattern is customizable, meaning it can be scaled up or down for use on large or tiny leaves, and the pattern can be printed using standard office printers. For durability, the printed pattern can be laminated or printed on a non-reflective hard board with the use of a transparent plastic cover to flatten the leaves for improved accuracy (Fig. 1c).

### 2.3 Feature extraction and leaf segmentation

**Fig. 2:**
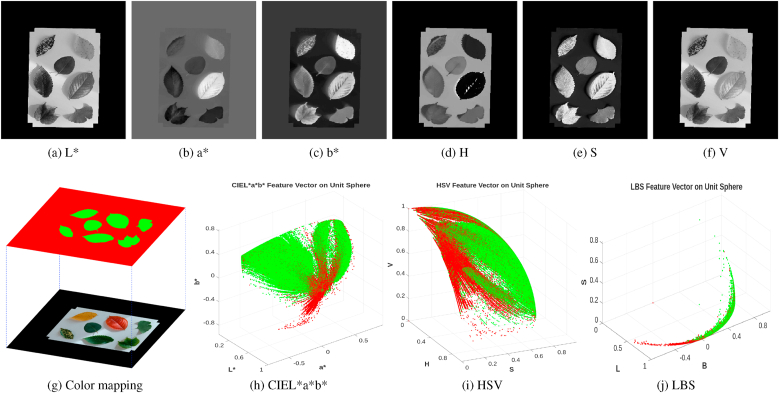
Comparison of different channels of CIEL∗a∗b∗ (a,b,c) and HSV color space (d,e,f) extracted from the sample image in Fig. 1d. The green-red-colored ground truth segmentation (g) is mapped into 3 different feature spaces: CIEL∗a∗b∗ (h), HSV (i) and LBS (j).

Extracting the most reliable features of the leaves is crucial for leaf segmentation. Many existing methods rely on intensity-based thresholding [37]. While these methods work if there is strong contrast between the leaves and the background in the image, they are not robust to shadows and illumination variations. In the field of leaf segmentation, the CIEL∗a∗b∗ color space [20] and HSV space [18,38] are often used instead of the RGB space, because the RGB space cannot distinguish the subtle differences between similar colors within the same spectrum. Through empirical analysis, we observed that the L∗ and b∗ channels of the CIEL∗a∗b∗ color space and the S and V channels of the HSV color space can effectively differentiate leaves from the background for most common leaf colors, while the a∗ channel and the H channel do not provide a consistent distinction. To illustrate this, we extracted the three channels of each color space from the sample image (Fig. 1d) and displayed them in Fig. 2. As shown, in the L∗ and V channels, the leaves appear consistently darker than the background, while in the b∗ and S channels, the leaves appear consistently brighter. In contrast, the a∗ and H channels show inconsistency — some leaves appear darker than the background, while others appear brighter. This is because the *a*∗ channel measures the green-red component of a color; the bigger the value is, the redder the color is. Since some leaves are green and some are red in the image (which is a general case), they show opposite values in the a∗ channel. Given that leaf colors are usually in the range between red and green, we thus cannot use the a∗ channel for leaf segmentation. The H channel is not a good choice either, because the hue value can be random in very dark areas (shadows) and very bright areas (pattern background). The L∗ and V channels both measure the lightness level, but the L∗ channel displays higher contrast (Fig. 2a and f). Therefore we combine the L∗ and b∗ channels of the CIEL∗a∗b∗ and the S channel of the HSV color space to form a new feature which we call the Leaf Background Separation (LBS) feature.

The LBS feature can accurately distinguish between leaf and a white background. To demonstrate this, we used the Segment Anything in High Quality Model (HQ-SAM) [45] to extract the ground truth mask of the leaves. We then created a color map (Fig. 2g), where foreground pixels are shown in green and background pixels in red to enhance visual contrast. Next, we extracted three types of feature vectors ($\vec{{\text{CIEL}}^{*}a^{*}b^{*}}$, $\vec{\text{HSV}}$, and $\vec{\text{LBS}}$) for each pixel in the original RGB image and visualized their distributions on a unit sphere (Fig. 2h–j) using the color map (Fig. 2g).

In both the CIEL∗a∗b∗ and HSV feature spaces, foreground (green) and background (red) pixels appear more interlaced, making separation less distinct. In contrast, in the LBS feature space, pixels are mapped to a curved band, with foreground pixels clustering at one end and background pixels at the other. This unique feature enables effective segmentation using angular distance. Although a small number of pixels are interlaced, they can be easily removed during post-processing using standard connected component morphological operations. Other features may also be used, such as texture features, edge features and chromatic features, however, we find that the LBS feature is the simplest and most effective for most general cases.

The leaf segmentation consists of four main steps:

1. Image cropping: crop the image and only keep the pattern region (excluding the fiducial markers);
2. Feature extraction: extract the LBS feature of the cropped region, and normalize all channels to the range [0,1];
3. Region segmentation: segment the cropped region into 2 regions (leaf and background) based on the extracted feature using K-means++ clustering algorithm [46] and angular distance:

(1) $$d\left(X,C\right)=1-\frac{X\cdot C^{\prime }}{\left\|X\|\cdot \|C\right\|}$$

where X is the LBS feature vector and C is the cluster center.

4. Leaf region determination: leaf region is determined by comparing the convex area of the 2 segmented regions — the one with a smaller convex hull area is the leaf region. Note that the convex hull area of the leaf region is always smaller than that of the background region unless we deliberately cover all the 8 extrema points on the pattern with leaves (please refer to Fig. S1 in the supplemental material file for detailed illustration).

At Step 2, additional features can be extracted and concatenated to the LBS feature to enhance segmentation reliability, such as texture features and color specific features. These are optional features on Leaf Analyzer which can be selected in case of need, though the LBS feature is sufficient in most cases. Additionally, concatenating too many features will slow down the segmentation speed.

At Step 3, we use angular distance instead of Euclidean distance because angular distance is scale-invariant and more effective for high-dimensional feature vectors. This is especially important when concatenating multiple features for robust segmentation.

### 2.4 Traits calculation

Once the leaf regions are segmented, traits such as area, perimeter, and count are derived from connected component properties. For the dimensions of the leaf, we use the algorithm described in Ref. [47] to compute the oriented bounding box which defines the width and length of the leaf. Basically, the algorithm computes the Feret diameter for a number of directions, and keeps the direction that produces the minimum-area enclosing rectangle (Oriented bounding box). For an illustration of the Feret diameter of a leaf, please refer to the supporting information (Fig. S2).

### 2.5 Text detection and recognition

Text recognition in Leaf Analyzer is performed in two steps: first, the text region in the image is detected using a pretrained Character Region Awareness for Text Detection (CRAFT) deep learning model; then, optical character recognition (OCR) is applied to extract the text. Since text recognition can be challenging and is not the primary focus of Leaf Analyzer, we do not aim to detect or recognize difficult cases such as cursive handwriting or special characters. However, for well-written or printed text, Leaf Analyzer can accurately recognize and process it without issues. It is important to note that adding text to the image or pattern is not required for Leaf Analyzer to function, but when present, it can be automatically recognized.

### 2.6 Evaluation of Leaf Analyzer

We evaluated the performance of Leaf Analyzer in terms of accuracy, precision and processing speed. Accuracy denotes how close a measured value is to the true value, while precision indicates the consistency or repeatability of measurements. We conducted the evaluation with Leaf Analyzer version 2.5. All the images used in the evaluation have been published on our Github repository (https://github.com/squashking/Leaf-Analyzer).

#### 2.6.1 Accuracy

Since obtaining the ground truth leaf area for real plants is challenging, it is standard practice to use geometric shapes with known areas for validation, as demonstrated in studies on LeafByte [37] and Leaf-IT [33]. For quantitative evaluation, we assessed Leaf Analyzer's accuracy in measuring the area of green circles and squares with known dimensions, using Absolute Percentage Error (APE) and Mean Absolute Percentage Error (MAPE) as performance metrics:

(2) $$APE=\frac{\left|M-G\right|}{G}\cdot 100\%$$

(3) $$MAPE=\frac{1}{n}\overset{n}{\underset{i=1}{\sum }}\frac{\left|M_{i}-G_{i}\right|}{G_{i}}\cdot 100\%$$

where M is the measurement from Leaf Analyzer, G is the ground truth area, and n is the total number of objects. We compared the results with Petiole Pro (a commercial software tool) and LeafByte (an open source tool). To ensure fairness of comparison, we take images with both our pattern and the competitor's pattern in the same image and use exactly the same image as input. We also compared the segmentation results of real leaf images taken indoors and outdoors with various lighting conditions, though the ground truthed values of these leaves is unknown.

#### 2.6.2 Precision

We printed our pattern and a green circle of known area on a piece of A4 paper and took 27 images from different heights and angles using an iPhone13 Pro Max camera. More specifically, we first kept the phone level with the paper and varied the height of the phone from 100 cm down to 30 cm and took an image every 5 cm (15 images in total). Next we kept the distance from the camera to the paper at roughly 50 cm while changing the angle of the phone camera from 90° to 35° and took an image every 5° (12 images in total). We then measured the area of the circle in the images using Leaf Analyzer, denoted the precision in the form of mean (*μ*) ± standard deviation(*σ*), and calculated the Coefficient of Variation:

(4) $$CV\%=\frac{\mu }{\sigma }\times 100\%$$

where *μ* is the mean of the measurements and *σ* is the standard deviation.

## 3 Results

### 3.1 Area measurement accuracy comparison with Petiole Pro

**Fig. 3:**
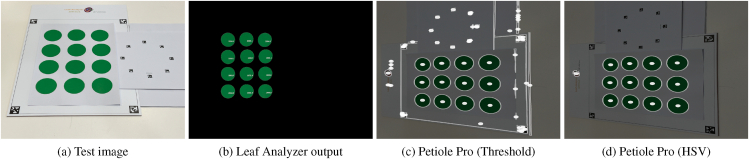
Accuracy of Petiole Pro and Leaf Analyzer comparison: (a) Test image; (b) Leaf segmentation and area estimation with Leaf Analyzer; (c) Leaf segmentation with Petiole Pro using the threshold method; (d) Leaf segmentation with Petiole Pro using the HSV method.

In this comparison experiment, we designed 12 green circles (simulating leaves) with a known area of 2000 mm^2^ using Inkscape [48] and printed them on A4 paper. The printed paper was placed on our pattern board (pattern size: 287 × 340 mm, AprilTag length: 20 mm), alongside the standard Petiole Pro pattern. We then captured an image of the paper from an angle using an iPhone 13 Pro Max to test geometric correction (Fig. 3a). As shown in Fig. 3, the circles can be successfully segmented on both Leaf Analyzer and Petiole Pro. Note that although the Leaf Analyzer and Petiole Pro patterns overlap in the image, Leaf Analyzer can automatically exclude the Petiole Pro pattern and the paper edges during processing. This allows the same image to be used as input for both tools without the need for cropping. It is the same for other comparison experiments described in Sec. 3. On Petiole Pro, there are two methods for leaf segmentation: the threshold method and the HSV method. The threshold method failed to fully remove the paper edges and board background, whereas Leaf Analyzer effectively masks out all unwanted background even though part of the Petiole Pro pattern overlaps the Leaf Analyzer pattern area. Note that the images are shown rotated by −90° on Petiole Pro because it does not read EXIF metadata of the image file (though this does not affect the measurements).

**Fig. 4:**
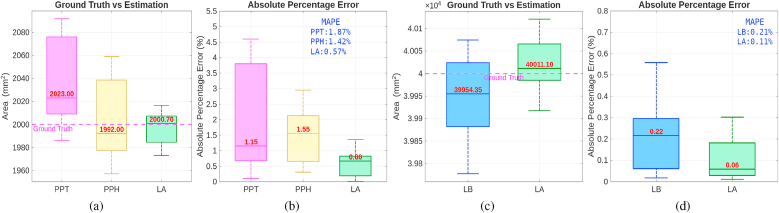
Accuracy comparison between Petiole Pro-Threshold (PPT), Petiole Pro-HSV (PPH), LeafByte (LB) and Leaf Analyzer (LA). The median values are marked in red. (a) Area estimation comparison with Petiole Pro. (b) Absolute percentage error comparison with Petiole Pro. (c) Area estimation comparison with LeafByte. (d) Absolute percentage error comparison with LeafByte.

Fig. 4a–b shows a comparison of the accuracy of measurement of the 12 green circles. Detailed measurements of the circle areas using the two software tools can be found in the supplemental material (Table S1). With Leaf Analyzer, the area of each circle can be computed in a single run, whereas Petiole Pro requires manual selection of each circle for individual measurement display. We can see that most of the measurements from Leaf Analyzer are closer to the ground truth than either method on Petiole Pro, with a median measurement of 2000.70, resulting in an error of only 0.70 mm^2^ (or 0.035 %), compared to an error of 23.00 mm^2^ (or 1.15 %) with Petiole Pro-Thresh method and 8.00 mm^2^ (or 0.4 %) with Petiole Pro-HSV method. Both Petiole Pro methods have a wider spread than Leaf Analyzer, indicating higher variability and inconsistency. The Petiole Pro-Threshold method shows an upward bias (above ground truth), as many of its measurements exceed the ground truth. The Petiole Pro-HSV method, on the other hand, has a downward bias (below ground truth) and the lowest median value among the three methods. The interquartile range (IQR) for both Petiole Pro methods is larger than that of Leaf Analyzer, suggesting that Leaf Analyzer provides the most consistent and reliable measurements. In addition, Leaf Analyzer achieves an MAPE of just 0.57 % while the MAPE of Petiole Pro-Threshold is more than 3 times higher (1.87 %) and Petiole Pro-HSV is almost 2.5 times higher (1.42 %). The measurements for each circle can be found in the supplementary file. It is clear that both Petiole Pro and Leaf Analyzer can perform geometric correction using their patterns. However, Leaf Analyzer achieves higher accuracy and precision.

### 3.2 Area measurement accuracy comparison with LeafByte

For comparison with LeafByte, we followed a similar approach as in Sec. 3.1, this time using a single green square (200 × 200 mm) since LeafByte cannot measure multiple leaves in one image. A pattern for Leaf Analyzer (260 × 310 mm, AprilTag length: 20 cm) and LeafByte (250 × 250 mm) was designed using Inkscape, with the LeafByte pattern embedded inside the Leaf Analyzer pattern. The pattern was printed on A3 paper, and the green square was randomly positioned within the pattern area. We captured 12 images from various angles and distances using an iPhone 13 Pro Max. Please refer to the supplemental material (Fig. S3) for a sample image and a comparison of area estimation accuracy between the two software tools, and to Table S2 for detailed measurements of the green square areas using both tools. We computed the estimated area and Mean Absolute Percentage Error (MAPE) using both software tools and show them in Fig. 4c–d.

With Leaf Analyzer, the area of the green square in 12 images can be calculated in one run by batch processing while on LeafByte, the images must be manually processed one by one. Also, on LeafByte we need to manually select the dot pattern because the software requires the image to contain only the dot pattern and the leaf. In contrast, Leaf Analyzer automatically masks out the LeafByte pattern and handles background clutter effectively. Fig. 4c–d compares the measurement accuracy across all 12 images. Leaf Analyzer exhibits a median value (40011.10 mm^2^) very close to the true area (40000 mm^2^), with a measurement error of only 11.10 mm^2^ (or 0.03 %) and a tight IQR, indicating high accuracy and consistency. In contrast, LeafByte shows a median (39954.35 mm^2^) slightly below the true area with a measurement error of 45.65 mm^2^, with a wider IQR, suggesting greater variability and a tendency to underestimate the area in some cases. While both tools perform reasonably well (APE <1 %), Leaf Analyzer has a lower MAPE compared to LeafByte (0.11 % vs. 0.21 %, Fig. 4d) and stands out for its precision and consistency. This experiment further reinforces Leaf Analyzer's reliability for accurate and consistent leaf area measurements.

### 3.3 Leaf segmentation accuracy comparison

To assess leaf segmentation accuracy, we collected real leaves of various colors and shapes from the Australian National University (ANU) Canberra campus. Images were captured using a cellphone both indoors and outdoors (simulating field conditions) with the three patterns (Leaf Analyzer, Petiole Pro, LeafByte) in the background under different lighting conditions. To ensure fairness of comparison, the exact same images were used as input for all three methods, without applying any method-specific image processing such as cropping. Leaf Analyzer was evaluated with the proposed LBS feature alone, without supplementary features.

#### 3.3.1 Leaf segmentation accuracy comparison with Petiole Pro

**Fig. 5:**
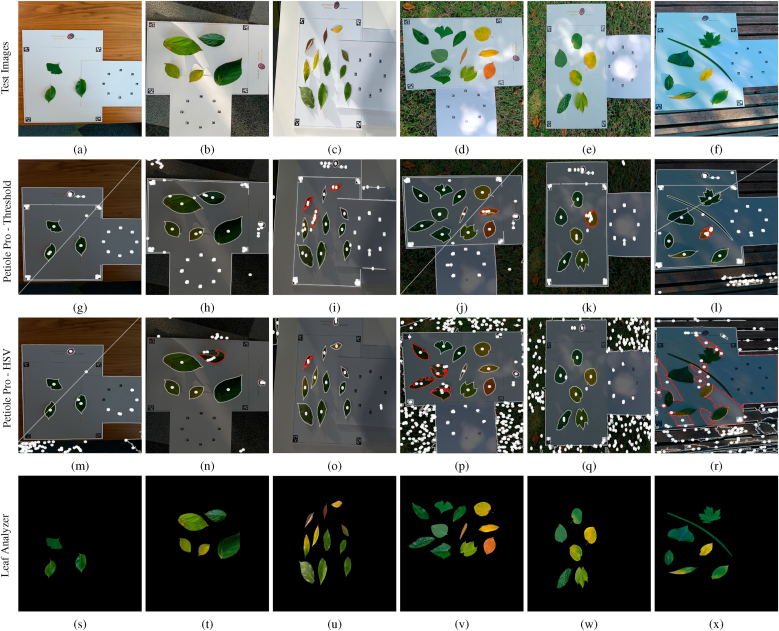
Leaf segmentation comparison. First row (a-f): Test images. 2nd row (g-l): Leaf segmentation using the threshold method on Petiole Pro. 3rd row (m-r): Leaf segmentation using the HSV method on Petiole Pro. 4th row (s-x): Leaf segmentation using Leaf Analyzer. The red curves marks the leaves that are not accurately segmented.

We captured six images of leaves of various shapes and colors under different lighting conditions with both the Petiole Pro and Leaf Analyzer pattern in the background (Fig. 5). The first three images were taken indoors while the remaining three images were taken outdoors to simulate field conditions. Fig. 5 shows screenshots of the leaf detection/segmentation results using Petiole Pro (2nd row: using threshold method; 3rd row: using the HSV method) and Leaf Analyzer (4th row). Petiole Pro annotates the detected leaf margins with white curves, and we highlighted incorrectly detected margins in red. When the leaves have similar colors and there are no heavy shadows, all three methods perform well (first column of Fig. 5). However, in challenging situations where the leaves have varying colors and there are illumination variations (Fig. 5b ∼ 5f), the two Petiole Pro methods struggle, while Leaf Analyzer consistently segments leaves accurately. In some instances (Fig. 5b and e), one Petiole Pro method succeeds while the other fails, and some leaves have multiple detections, demonstrating that relying solely on intensity features (threshold method) or color features (HSV method) is unreliable. The LBS feature is more robust because it integrates both intensity and color information. Overall, the HSV method performed most poorly (e.g., in Fig. 5r, no leaves were correctly detected) in challenging lighting situations, as color features are highly sensitive to illumination changes. These results demonstrate the robustness of Leaf Analyzer under varying lighting conditions, making it well-suited for field applications.

#### 3.3.2 Leaf segmentation accuracy comparison with LeafByte

**Fig. 6:**
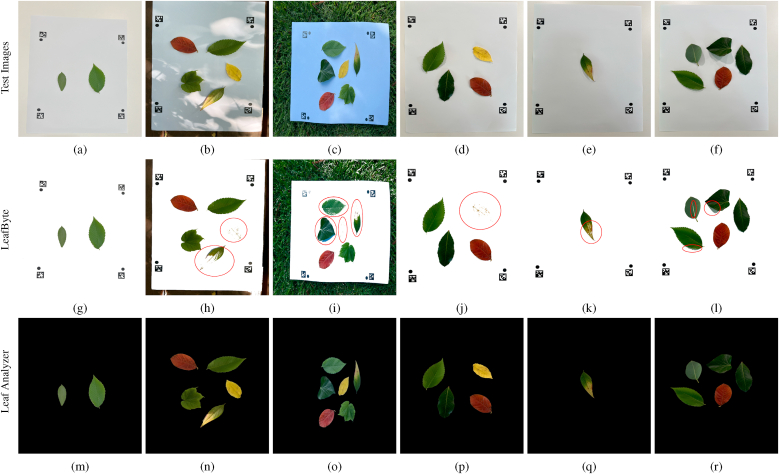
Leaf segmentation comparison. First row (a-f): input images. 2nd row (g-l): leaf segmentation using LeafByte (the red circles mark inaccurately segmented leaves). 3rd row (m-r): leaf segmentation using Leaf Analyzer.

To compare leaf segmentation performance with LeafByte, we printed both patterns on the same paper and put leaves on it. We took six images (Fig. 6); two of them taken outdoors (Fig. 6b–c) and the rest taken indoors with varying lighting conditions. LeafByte performs well under shadowless conditions with strong contrast between the leaf and the background. However, its accuracy decreases when the background contains colors other than white (Fig. 6b–c) due to its reliance on Otsu's method [19], which fails when the histogram of luminance values is not bimodal. But Leaf Analyzer does not have such restrictions because any background beyond the pattern area will be cropped out. Additionally, LeafByte fails to segment yellow leaves (Fig. 6h–j). Therefore if a leaf is partially yellow (e.g., a diseased leaf), the segmentation will not be accurate (Fig. 6k). Also we notice that LeafByte struggles when leaves have lighter-colored regions, such as the vein of one of the leaves in (Fig. 6l). In all six test images, Leaf Analyzer demonstrates precise segmentation with sharp and clear margins, unaffected by background colors or leaf color variations.

### 3.4 Precision of Leaf Analyzer

**Fig. 7:**
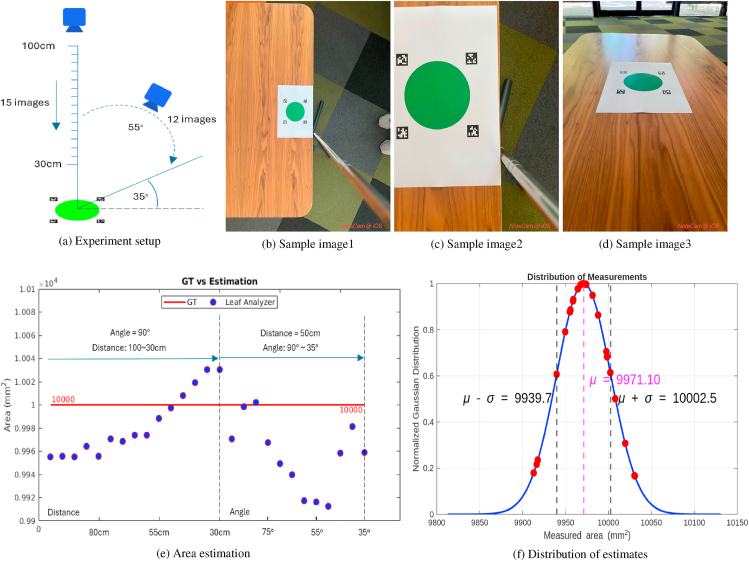
Precision test setup and results. (a) Experimental setup. (b) Image taken at distance: 100 cm, angle: 90°. (c) Image taken at distance: 30 cm, angle: 90°. (d) Image taken at distance: 50 cm, angle: 35°. (e) Twenty-seven measurements acquired under different camera heights and angles compared against the ground truth. (b) Distribution of the measured values.

To evaluate the precision of Leaf Analyzer, we printed a test pattern (size: 140 × 140 mm, tag length: 20 mm) and a green circle (area: 10000 mm^2^) on A4 paper and captured 27 images as described in Fig. 7a–d. The images were taken using an iPhone 13 Pro Max with the NoteCam app, which records both GPS coordinates and pitch angle information. First we kept the camera at a right angle with the paper and gradually lowered the distance from 1 m to 30 cm, taking an image every 5 cm (15 images in total). Then we kept the distance at 50 cm but varied the angle from 90° to 35° and took an image roughly every 5° (12 images in total).

Fig. 7e–f illustrate the estimated circle area and the distribution of the estimates. We can see from Fig. 7e that as the distance of the camera goes down, the estimate first gets closer to the ground truth till the distance reduces to 50 cm, when the estimate is the closest to the ground truth. Beyond this point, the estimate starts to get further from the ground truth as the distance gets too small. When the camera angle is tilted from 90° to 35°, the estimates follow a U-shaped trend: they initially deviate from the ground truth, reach the maximum deviation at around 50°, and then gradually return closer to the ground truth. It is important to note that precise equipment was not used to control camera orientation or distance, so the recorded experimental conditions may have some inaccuracies. The results also indicate that changing the camera angle can lead to greater deviations from the ground truth compared to changing the camera distance. This is because images inevitably have distortions that cannot be fully corrected without camera calibration, which is not applicable to general software tools like Petiole Pro, LeafByte, and Leaf Analyzer. However, the deviations remain minimal due to geometric correction. From the test data, we obtained a distribution with a mean area of 9971.1 mm^2^ and a standard deviation of 31.4. Thus, the precision of measurement is specified as 9971.1 ± 31.4 mm^2^. If we normalize this result with the standard deviation using Eq. (4), we get a precision of 1 ± 0.31 %. Note that since the images were not taken under optimal conditions, in real applications we can expect to get a much better precision.

### 3.5 Processing speed of Leaf Analyzer

The runtime of Leaf Analyzer depends on several factors, including image resolution, the set of traits selected for measurement, the number of leaves per image, whether leaf masks are saved, and the computer hardware and operating system. In general, higher-resolution images and a larger trait set increase processing time.

When working with very high–resolution images, downsampling can substantially improve speed with little impact on accuracy for most morphological traits. The resizing factor can be configured under the *Advanced* tab in the *Settings* panel. In our precision experiment (Sec. 3.4), the 27 test images were processed at half of their original resolution (3024 × 4032; 12.2 MP) and still achieved high precision. In that experiment, we computed only the total leaf area per image and exported results to Excel using the batch-processing feature. The full set of 27 images completed in 35.06s (approximately 1.30s per image) on our computer (Intel® Xeon® Silver 4112 × 16, Ubuntu 24.04).

## 4 Discussion

### 4.1 Fully automated leaf trait measurement

One of the key features of Leaf Analyzer is its ability to perform leaf trait measurements fully automatically, requiring no human interaction during runtime. Users only need to select the image(s), choose the traits to measure (or use the default traits), input the dimensional parameters of the pattern, and click the “Run” button. The results are then saved in a spreadsheet file for easy access. By “fully automated,” we mean that no manual intervention — such as parameter tuning — is needed during the execution of the measurement process. To demonstrate its functionality, we have prepared two demo videos as supplemental material: one for leaf morphological trait measurement (Youtube link or Youku link) and another for leaf damage assessment (Youtube link or Youku link).

The step of inputting dimensional parameters could also be automated in future versions, for example, by encoding the parameters in a QR code or printing them directly on the pattern and recognizing them through optical character recognition (OCR). However, to keep the pattern simple and easy for users to customize and print themselves, we currently require manual input. Additionally, if all images to be analyzed use the same-sized pattern, the user only needs to input the parameters once, minimizing repetitive tasks.

The unsupervised leaf segmentation algorithm is designed to work effectively with minimal user intervention. It has very few adjustable parameters, and the default settings are sufficient for most use cases. This ensures ease of use while maintaining robust performance across a wide range of conditions.

### 4.2 Effectiveness of Leaf Background Separation (LBS) feature in segmenting complex leaf colors

The Leaf Background Separation (LBS) feature plays a pivotal role in automatic leaf segmentation. This feature leverages the fact that most leaves exhibit colors within the red-to-green spectrum, as discussed in Sec. 2.3. While alternative color features such as CIEL∗a∗b∗ and HSV can also be used, they are more susceptible to failure under uneven lighting conditions or when leaf colors are heterogeneous.

**Fig. 8:**
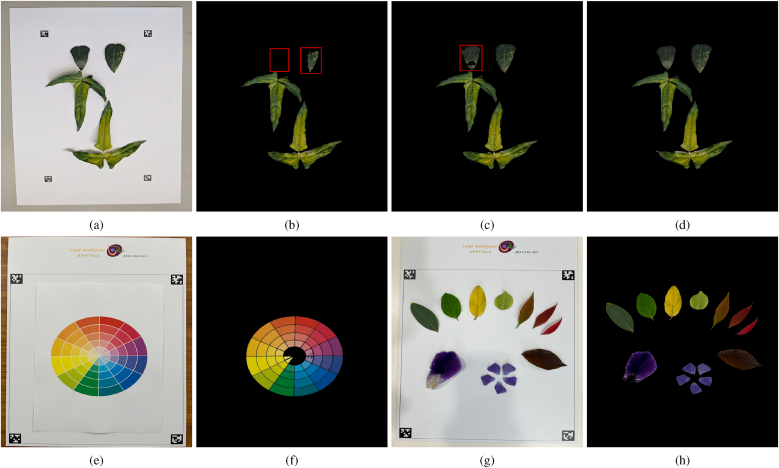
**Top row**: comparison of leaf segmentation using CIEL∗a∗b∗, HSV, and LBS features — (a) Original RGB image of cowpea leaves, (b) segmentation result using CIEL∗a∗b∗ feature, (c) segmentation result using HSV feature, and (d) segmentation result using the proposed LBS feature; red boxes indicate segmentation errors. **Bottom row**: tests across the full visible spectrum — (e–f) printed color wheel and its LBS segmentation; (g–h) real leaves/petals spanning diverse colors and their LBS segmentation.

The top row in Fig. 8 presents an example of segmenting cowpea leaves using CIEL∗a∗b∗, HSV, and LBS features. In this example, the top two unifoliate leaves emerging after cowpea germination are very dark green, whereas the two trifoliate leaves display a mixture of green and yellow hues. The a∗ channel in the CIEL∗a∗b∗ space fails to distinguish between the leaves and the background, resulting in one dark leaf being entirely missed and another being only partially segmented (Fig. 8b). The HSV-based method performs slightly better but still struggles to segment one of the dark leaves accurately, as the hue component does not sufficiently differentiate between the leaf and the background. In contrast, the LBS feature integrates the most discriminative elements from both the CIEL∗a∗b∗ and HSV color spaces, producing a more accurate segmentation result (Fig. 8d).

While LBS is especially robust for red–green foliage, it also works beyond this range because its three components provide complementary evidence in the K-means++ feature space (with angular distance). One may intuitively think of this as a ‘voting-like’ effect — without implying a literal voting scheme — where reduced discriminability in the b channel can be offset by informative L and S cues under reasonable illumination. To verify this, we imaged a printed color wheel spanning the visible spectrum on the Leaf Analyzer pattern board (Fig. 8e); LBS correctly segmented nearly all color blocks (Fig. 8f), with expected failures only near the desaturated center where colors approach the white background. We further tested real leaves and *petals* spanning a wide color range (Fig. 8g); despite moderate shadows, LBS segmented the objects (including purple pedals) well (Fig. 8h). The lower portion of the purple petal on the left is not fully segmented because its chroma is close to the background — an acknowledged limitation when foreground saturation is very low.

### 4.3 More applications of Leaf Analyzer

**Fig. 9:**
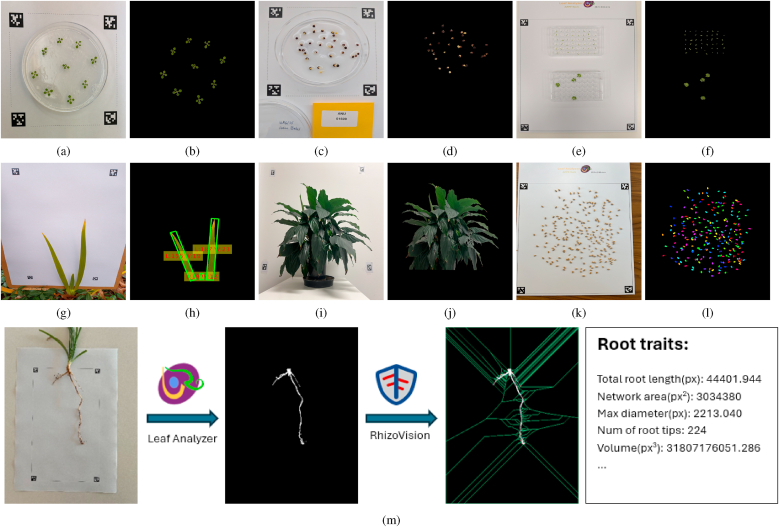
More applications of Leaf Analyzer. (a–b): Petri dish plant leaf area measurement. (c–d): Petri dish seed germination monitoring, (e–f): 48-well plate hydroponic plant leaf area measurement. (g–h): Nondestructive leaf area and dimensions measurement. (i–j): Silhouette leaf area measurement. (k–l): Seed counting. (m): An example root phenotyping pipeline using Leaf Analyzer.

Leaf Analyzer can be used for nondestructive leaf trait measurement and a broad range of potential applications. Since it can measure morphological traits of any non-white objects, Leaf Analyzer can be used to measure the projected area of plant flowers, pods, seeds, root systems, and other plant structures (Fig. 9). Below, we outline some additional applications of Leaf Analyzer.

#### 4.3.1 Versatile nondestructive measurements

Leaf Analyzer enables nondestructive measurement of leaf traits (Fig. 9a–j), meaning leaves can be measured *in situ* without detaching them from the plant. By placing a reference pattern behind the leaves and capturing an image, accurate measurements can be obtained without harming the specimen.

This approach is particularly useful for measuring the leaf area of small plants that cannot be assessed using large equipment such as Phenospex PlantEye. Fig. 9a–b illustrate how Leaf Analyzer can measure the leaf area of Arabidopsis growing in an agar-filled Petri dish. The Petri dish silhouette is automatically classified as background, eliminating the need to remove the plant for measurement. Similarly, seeds grown in petri dishes can be automatically segmented, enabling monitoring of germination by tracking changes in the area of each seed (Fig. 9c–d). This method is also applicable to plants grown in other transparent or white containers, such as 48-well plates (Fig. 9e–f).

For larger plants, one or more leaves can be measured at a time similar to the LI-COR Handheld Laser Leaf Area Meter. Fig. 9g–h shows an example of nondestructively measuring leaf dimensions using Leaf Analyzer. Furthermore, the scalable and customizable pattern design allows for measurements across a wide range of plant sizes—from small seedlings to large trees. For very large plants (e.g., Fig. 9i), the reference pattern can be affixed to a white wall with four AprilTags, enabling the measurement of projected silhouette or crown projection area, which can then be correlated with total leaf area [49]. In this configuration, a direct measurement of projected silhouette area with Leaf Analyzer will not be accurate because the plant and pattern are not coplanar. However, if the distances from the pattern plane and from the plant to the camera are both kept constant when fitting a linear or polynomial regression, the model implicitly accounts for the bias, since the measured silhouette area is scaled by a constant factor (see Appendix A for a rigorous derivation). If an accurate projected silhouette area is required, the measurement can be corrected using Eq. A.6.

For more non-destructive measurement examples, please refer to Fig. S4 in the Supplemental Material.

#### 4.3.2 Seed counting

Leaf Analyzer's ability to segment any non-white object placed within the calibration pattern can be repurposed for fast, reliable seed counting (Fig. 9k–l). Seeds can be dispersed within the pattern region to acquire an image; the software then segments seeds in much the same way it processes leaves. When seeds are spaced apart, counting is straightforward — each segmented object corresponds to a single seed. In cases where seeds are touching or clustered, Leaf Analyzer includes a built-in watershed-based separation method [50] to resolve overlapping objects. This functionality can be enabled by selecting the “Watershed” option under the *Advanced* tab in the *Settings* panel. This feature ensures accurate seed counts even under challenging imaging conditions, supporting rapid, automated analysis in seed phenotyping and quality control applications.

#### 4.3.3 Root phenotyping

Measuring root traits can be challenging due to the fine structure and complex coloration of roots, which pose challenges for segmentation using conventional tools like ImageJ. Automatic root segmentation methods typically involves training a model on a large dataset (e.g., the Root Phenotyping Toolbox [51]) or through corrective annotation (e.g., Root Painter [52]), which is very time-consuming. However, if the roots can be extracted from the soil, they can be analyzed using a combination of Leaf Analyzer and RhizoVision [53]. Leaf Analyzer can generate an accurate binary mask of the roots, which can then be imported into RhizoVision for detailed trait analysis. Fig. 9m illustrates a root phenotyping pipeline using this approach.

#### 4.3.4 Leaf damage assessment

**Fig. 10:**
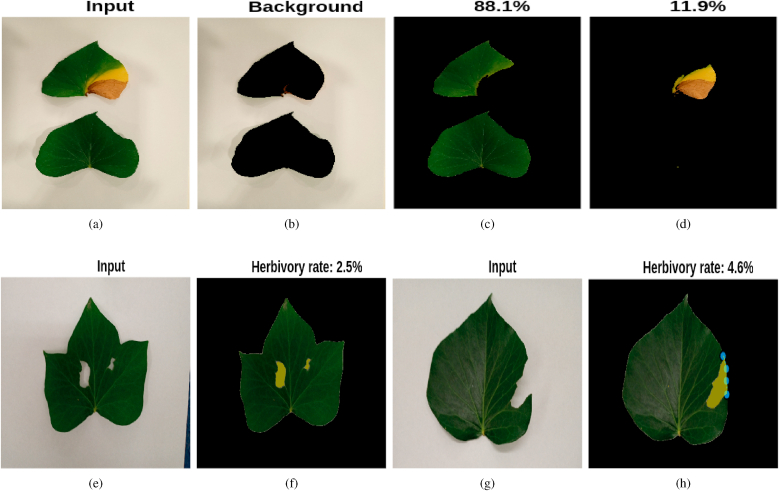
Leaf damage assessment using Leaf Analyzer. **Top row**: leaf diseased percentage measurement; the input image (a) is segmented into 3 parts: (b) The background (c) The healthy part (d) The diseased part. **Bottom row**: leaf percent herbivory measurement. Leaf grazed in the middle (e) and the computed herbivory rate (f). Leaf grazed on the margin (g) and the computed herbivory rate (h).

Leaf damage can result from various factors, including disease and herbivory. These two forms of damage manifest differently: disease-induced damage typically results in color changes while maintaining leaf integrity, whereas herbivory leads to missing leaf portions. Leaf Analyzer can quantify both types of damage by leveraging the same segmentation algorithms discussed in Section 2.3. For disease-induced damage, the segmentation algorithm must classify the leaf into three distinct regions: background, healthy tissue, and diseased tissue (Fig. 10a–d).

For herbivory assessment, if the damage occurs within the leaf, the percent damage can be directly computed (Fig. 10e–f). However, if the damage affects the leaf margins, manual drawing of the margin is required, similar to the method used in Ref. [37] (Fig. 10g–h).

### 4.4 Methods to improve the measurement

#### 4.4.1 Flattening the leaves

**Fig. 11:**
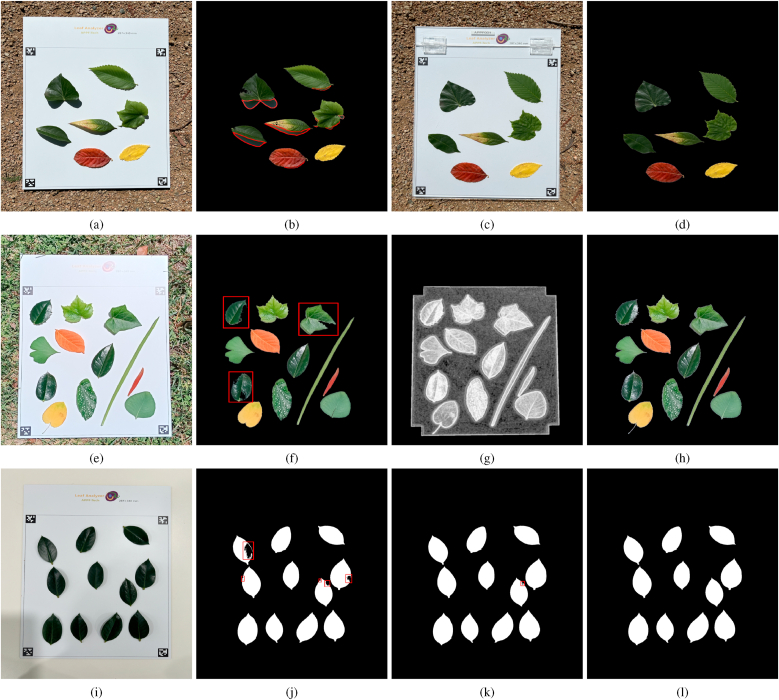
Improving segmentation with leaf flattening and incorporating additional features. **Top row (a**–**d):** Flattening reduces shadow artifacts — (a) unflattened leaves with severe shadowing; (b) segmentation of (a) showing shadow leakage (or shadow-induced false positives, marked with red outlines); (c) flattened leaves under a transparent cover; (d) improved segmentation after flattening. **Middle row (e**–**h):** Handling glare — (e) sunlit image with strong glare; (f) LBS segmentation; (g) local-entropy map; (h) LBS + entropy segmentation. **Bottom row (i**–**l):** Dark leaves under uneven lighting — (i) input image; (j) LBS segmentation binary mask with loss of peripheral leaf area; (k) LBS + DGCI segmentation binary mask; (l) LBS + DGCI + LoG segmentation binary mask, with further enhanced boundary completeness; red boxes indicate segmentation errors.

Leaves often exhibit natural curvature, which can reduce measurement accuracy if not accounted for. Flattening the leaves before imaging can significantly improve accuracy. There are many methods to flatten leaves. For example, one can use a flatbed scanner, a pair of transparent plastic clear file folder sheets [36], splints [5], acrylic boards [22], or a transparent glass plate [17]. Flattening not only improves measurement precision, but also reduces shadows, which can interfere with leaf segmentation. Fig. 11(a–d) illustrates a case where unflattened leaves with severe shadows fail to be segmented accurately (shadow borders are highlighted in red (Fig. 11b)). However, when the leaves are flattened using the proposed acrylic pattern board (Fig. 1c), the segmentation accuracy improves dramatically.

#### 4.4.2 Incorporating additional features

The proposed LBS feature works well for leaf segmentation in most cases. However, in some challenging situations, more features may need to be added to improve the segmentation. For example, in an uneven lighting setting, when images are taken from a certain angle, the leaves may have strong reflection. When glare is present, some parts of the leaf may lose color information, leading to incomplete segmentation. If partial color information remains, this issue can be mitigated by integrating additional features into the clustering algorithm. For example, texture features such as local entropy, local range, or local standard deviation can help distinguish leaves from the background even under difficult conditions. Fig. 11(e–h) shows an example of segmenting an image with strong glare using the LBS feature plus local entropy feature. Some leaves affected by glare were not well segmented using the LBS feature alone (Fig. 11f), and the issue can be resolved by combining the local entropy feature (Fig. 11h).

A second challenge is segmenting very dark (near-black) leaves under uneven lighting — an issue for color/intensity–based tools (e.g., Petiole Pro, LeafByte) and for LBS as well. In LBS, the B channel provides limited discrimination for nearly achromatic (black/white) regions; if strong shadows or glare simultaneously depress luminance (L) and saturation (S), LBS alone may under-segment margins. Fig. 11i–l illustrates dark-green leaves with uneven lighting: LBS alone leaves small missing segments along some borders (Fig. 11j). Accuracy improves by incorporating the Dark Green Color Index (DGCI) [54] (Fig. 11k), and improves further when LBS + DGCI are combined with a Laplacian-of-Gaussian (LoG) edge-enhancement feature to strengthen boundary contrast (Fig. 11l). In other scenarios, color-specific indices such as Excess Green (ExG) [55] (for green-dominant foliage) or High-Resolution Flower Index (HRFI) [56] (for yellow tones) can be beneficial. All of these additional features are implemented in Leaf Analyzer and can be enabled as needed.

### 4.5 Limitations of Leaf Analyzer

The experimental results demonstrate that Leaf Analyzer performs well in segmenting leaves of various colors and shapes, even under challenging lighting conditions. However, certain limitations should be acknowledged. First, the software requires leaves to be separated in the image; otherwise, overlapping leaves will be counted as a single entity, which is a common limitation to other software such as Petiole Pro and LeafByte. Second, because the background of the pattern is white, Leaf Analyzer is not effective for analyzing white leaves. Fortunately, white leaves are uncommon in most plant species. Additionally, while Leaf Analyzer does not require a strong contrast between the foreground and background — unlike some existing methods [34,37] — or a strictly perpendicular camera angle [5,33], extreme lighting conditions or highly skewed perspectives can still lead to segmentation errors or reduced measurement accuracy.

### 4.6 Comparison with deep-learning segmentation methods

Deep learning–based approaches (e.g., U-Net [6]/DeepLab [57]/Mask R-CNN [25]/MobileNet V3 [26] and recent transformer variants) excel at learning rich features under complex backgrounds and variable lighting, and are particularly strong for instance segmentation, which is advantageous when leaves overlap or occlude one another. Their trade-offs include the need for sizable, well-annotated training sets, sensitivity to domain shift (species, sensor, site), heavier compute and memory requirements, and reproducibility considerations tied to model versions and training procedures. In contrast, Leaf Analyzer uses an unsupervised clustering pipeline with the LBS feature, requires no labeled data or model training, runs efficiently on commodity CPUs, and offers transparent behavior with batch processing and optional features. Compared with deep-learning approaches, its limitations are that (i) it expects leaves on a white background with the calibration pattern; (ii) instance segmentation assumes leaves are separated—the watershed option can help when they touch, but accuracy is not guaranteed under heavy overlap; (iii) LBS relies on color/illumination cues, so severe shadows with chromaticity similar to leaves can degrade performance; and (iv) because clustering assumes the presence of both background and leaf pixels, extreme foreground–background imbalance can cause failures — e.g., when leaves cover nearly the entire pattern area or occupy only a tiny portion of it. A key advantage, however, is robust cross-species deployment without model training — Leaf Analyzer is not tailored to specific leaf shapes or colors and thus avoids common generalization issues seen in trained models. In practice, deep learning is preferable for dense, overlapping canopies and heterogeneous scenes when data and GPUs are available; Leaf Analyzer is well suited for rapid, training-free, high-throughput workflows with controlled backgrounds.

### 4.7 Comparison with 3D leaf-area measurement methods

Three-dimensional approaches — such as multi-view photogrammetry [27], structured-light scanning, and LiDAR — estimate true leaf surface area and can better handle curled or overlapping leaves and complex canopies. These methods are powerful when the biological question depends on surface geometry (e.g., boundary layer modeling, light interception) or when occlusion must be explicitly resolved. However, they typically require specialized hardware or controlled acquisition, careful calibration, meshing or parametric surface modelling [58], longer processing pipelines, and higher costs; throughput in field conditions can be constrained by wind, variable illumination, and occlusion. By contrast, Leaf Analyzer operates on single RGB images with a simple calibration pattern, delivering high-throughput, low-cost measurements of projected area and related traits on modest hardware. For use cases where projected silhouette or crown projection area is an adequate proxy for total leaf area, Leaf Analyzer can provide rapid estimates and, if needed, apply geometric corrections or empirical calibration to account for off-plane bias. Thus, 2D and 3D methods should be seen as complementary: 3D yields richer geometric quantification, while Leaf Analyzer emphasizes scalability, simplicity, and speed.

## 5 Conclusion

In this paper, we introduced Leaf Analyzer, a robust and efficient tool for leaf segmentation and morphological trait measurement. Unlike many existing methods that rely on strong contrast between the leaf and background or require strict imaging conditions, Leaf Analyzer employs a novel reference pattern and the Kmeans++ clustering algorithm with the Leaf Background Separation (LBS) feature to enable accurate segmentation under diverse lighting conditions and camera orientations. Our results demonstrate that the tool is capable of handling leaves of various shapes and colors with high accuracy, even in non-ideal imaging environments.

Despite its advantages, Leaf Analyzer has certain limitations. Leaves must be well-separated in the image to avoid being counted as a single entity, and white leaves cannot be processed effectively due to the white background of the reference pattern. Additionally, extreme lighting conditions may still impact segmentation performance. To address this limitation, we explored techniques such as leaf flattening and the incorporation of additional leaf features (e.g., texture features and color specific features), both of which noticeably enhance measurement accuracy.

Beyond leaf trait measurement, we demonstrated that Leaf Analyzer can be applied to a range of plant science applications, including nondestructive measurement, root phenotyping, and leaf damage assessment. The tool's flexibility allows it to be adapted for measuring other plant morphological traits, such as the projected area of plant flowers, pods, seeds or tree crown silhouettes.

Future work will focus on expanding the capabilities of Leaf Analyzer by integrating advanced machine learning techniques for improved segmentation, supporting more complex plant structures, and developing an automated workflow for large-scale plant trait analysis. We also plan to enhance the user interface to facilitate broader adoption by researchers and agronomists. By providing an accessible, accurate, and efficient method for leaf analysis, Leaf Analyzer has the potential to advance research in plant phenotyping, agronomy, and ecology.

## Author contributions

TH: investigation, conceptualization, methodology, software development, experiment design, formal analysis, and original manuscript. RP: validation, supervision, review and editing. DW: supervision, review and editing. All authors have read and approved the final manuscript.

## Data availability

The Leaf Analyzer source code, platform-specific installer files, and all data used in this study are publicly available on our GitHub repository at https://github.com/squashking/Leaf-Analyzer.

## Competing interests

The authors declare that they have no competing interests.

## Figures and Tables

**Fig. A.12:**
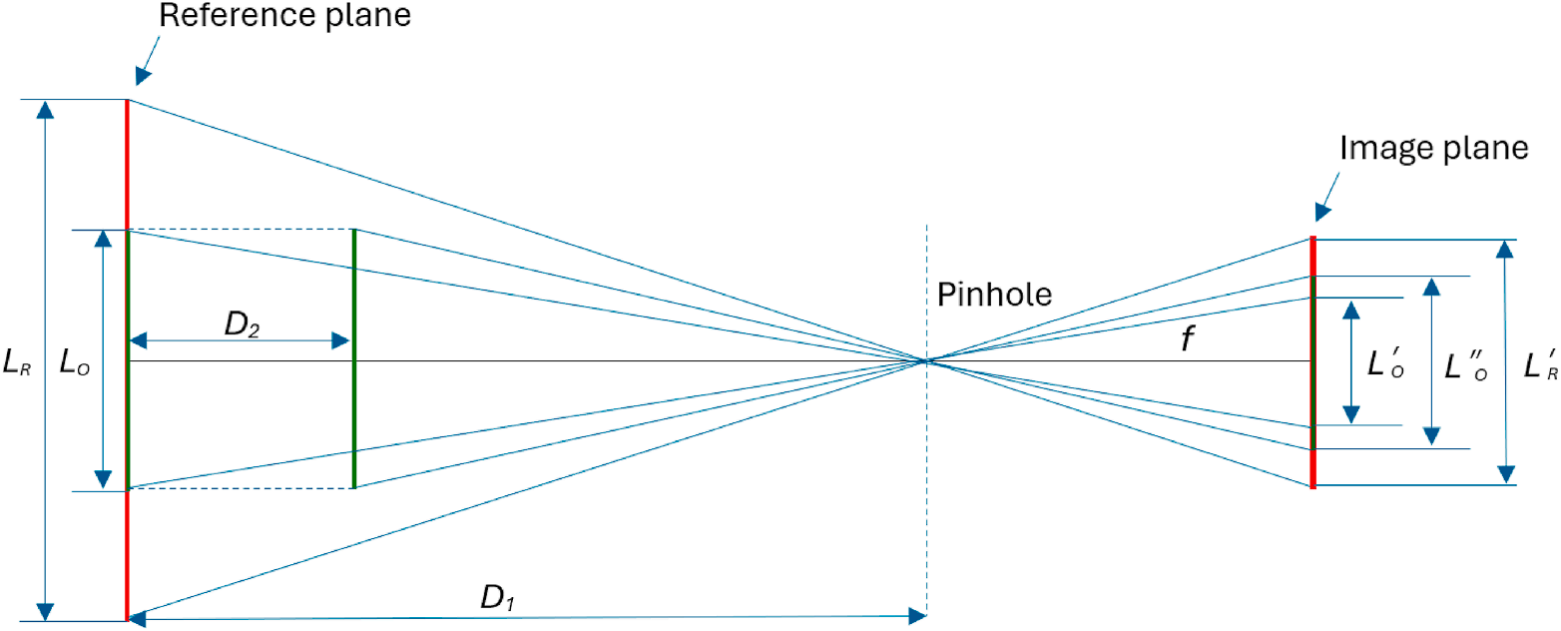
Pinhole-camera geometry for off-plane correction. Moving the object (green) a distance *D*_2_ toward the camera increases its projected length relative to the reference (red), which remains on the calibration plane; this induces a scale bias that can be corrected using Eqs. A.5–A.6.

## Appendix A Correcting measurements for off-plane objects

Leaf Analyzer assumes that leaves (or, more generally, objects) lie in the same plane as the reference pattern to ensure accurate scaling. When this is difficult to achieve (e.g., Fig. 9i), measurements can be adjusted to minimize error using a pinhole-camera model (Fig. A.12), which applies to most cameras used for plant phenotyping.

In Fig. A12, the green and red vertical segments on the left denote the object dimension to be measured and the reference length (e.g., a pattern dimension), respectively; their image projections appear on the right. If the object lies on the same plane as the reference, the image scale *S*_*I*_ is

(A.1) $$S_{I}=\frac{L_{O}^{\prime }}{L_{R}^{\prime }}=\frac{L_{O}}{L_{R}}=S_{T},$$

where *L*_*O*_ and *L*_*R*_ are the true object and reference lengths, $L_{O}^{\prime }$ and $L_{R}^{\prime }$ are their lengths in the image, and *S*_*T*_ is the true (scene) scale. Thus *S*_*I*_ = *S*_*T*_ when the object and reference are coplanar.

If the object is displaced toward the camera by a distance *D*_2_ along the optical axis (with camera–reference distance *D*_1_, so 0 ≤ *D*_2_ < *D*_1_), the object appears larger while the reference projection is unchanged. By similar triangles, the image scale becomes

(A.2) $$S_{I}^{\prime }=\frac{L_{O}\prime \prime }{L_{R}^{\prime }}=\frac{L_{O}}{L_{R}}\cdot \frac{D_{1}}{D_{1}-D_{2}}=S_{T}\cdot \frac{D_{1}}{D_{1}-D_{2}},$$

where $L_{O}\prime \prime$ is the new projected length of the object. If we directly use $S_{I}^{\prime }$ to measure object morphological traits, relative percentage errors then follow as

(A.3) $$E_{L}=\frac{D_{2}}{D_{1}-D_{2}}\cdot 100\%,$$

(A.4) $$E_{A}=\frac{2\cdot D_{1}D_{2}-D_{2}^{2}}{{\left(D_{1}-D_{2}\right)}^{2}}\cdot 100\%,$$

for length- and area-based traits, respectively. Equations A.3–A.4 show that error can be reduced by decreasing the misalignment *D*_2_ (bringing the object onto the pattern plane) or increasing *D*_1_ (imaging from farther away).

To correct measurements acquired with the off-plane scale in Eq. A.2, we can actually multiply the measurements by a factor. Let *L*_*I*_ and *A*_*I*_ denote length- and area-based measurements obtained using $S_{I}^{\prime }$. The corrected values are

(A.5) $$L_{C}=L_{I}\cdot \frac{D_{1}-D_{2}}{D_{1}}$$

(A.6) $$A_{C}=A_{I}\cdot {\left(\frac{D_{1}-D_{2}}{D_{1}}\right)}^{2}$$

where *L*_*C*_ and *A*_*C*_ are the corrected length- and area-based measurements, respectively. As expected, setting *D*_2_ = 0 yields *L*_*C*_ = *L*_*I*_ and *A*_*C*_ = *A*_*I*_. In practice, it is not necessary to measure *D*_1_ and *D*_2_ explicitly. If the imaging geometry is kept fixed—e.g., by mounting the camera on a tripod to hold *D*_1_ constant and positioning the object at a fixed offset *D*_2_—then the right-hand sides of Eqs. A.5–A.6 reduce to the measured quantities multiplied by a constant scale factor. This constant can be obtained via a simple one-time calibration:

1. Place a *planar calibration target* (e.g., a colored geometric shape) of known true area *A*_ref_ on the *same plane as the offset plant.*
2. Measure its area in Leaf Analyzer to obtain *A*_*I*,ref_.
3. Compute the calibration constant

$$C=\frac{A_{\text{ref}}}{A_{I,ref}}.$$

Once *C* is known, correct subsequent measurements acquired under the same setup as

(A.7) $$L_{C}=L_{I}\cdot \sqrt{C}$$

(A.8) $$A_{C}=A_{I}\cdot C$$
