# Supplementary material for: Leaf Analyzer: A fully automated and open-source tool for high-throughput leaf trait measurement
Source: Plant Phenomics. 2025 Dec 26;8(1):100145. doi: 10.1016/j.plaphe.2025.100145 (PMC13109305; doi:10.1016/j.plaphe.2025.100145)
Supplement: Multimedia component 1 [file mmc1.pdf]

Supplemental Material

Leaf Analyzer: A Fully Automated and Open-Source Tool for High-Throughput Leaf Trait Measurement

Tao Hu, Richard Poire, Danielle Way

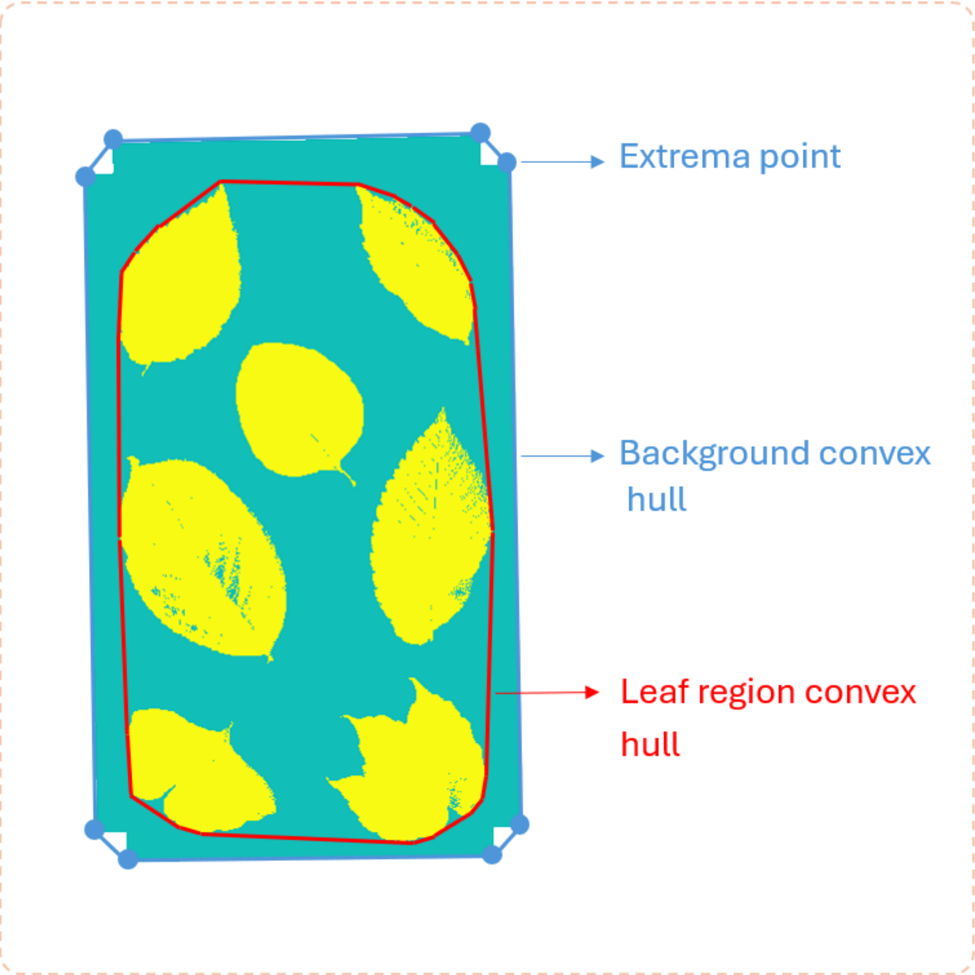

Figure S1: Illustration of convex hull. The convex hull of the background contains that of the leaf region, so the convex hull area of the background region is always larger than that of the leaf region unless all the 8 extrema points are covered by leaves.

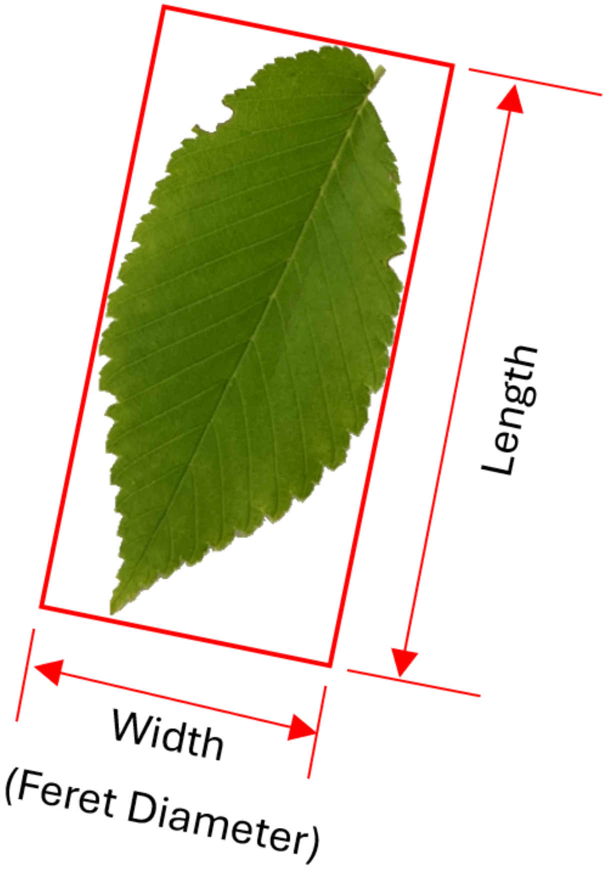

Figure S2: Illustration of the oriented bounding box method for leaf size estimation.

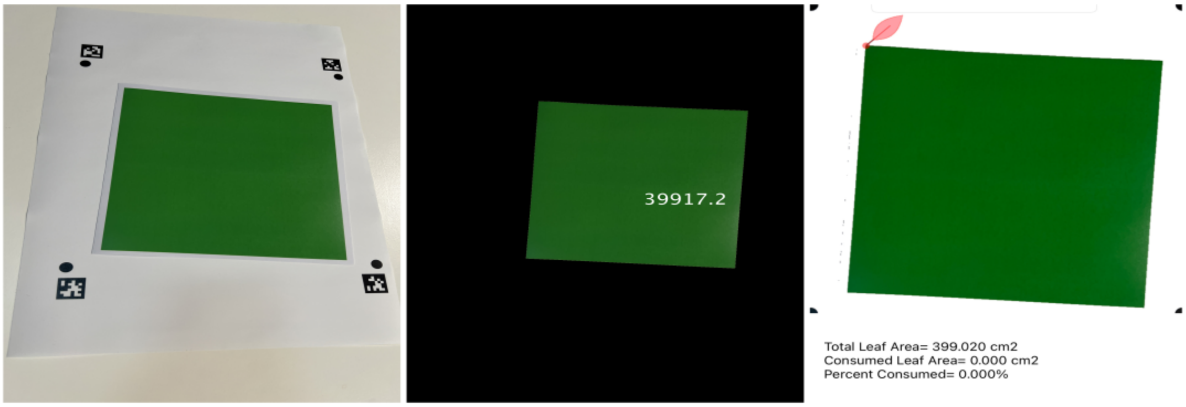

(a) One test image (b) Leaf Analyzer (c) LeafByte

Figure S3: Comparison of area estimation accuracy between LeafByte and Leaf Analyzer: (a) One of the 12 test images, (b) Leaf segmentation and area estimation with Leaf Analyzer, (c) Leaf segmentation and area estimation with LeafByte. For all other test images, please go to our Github repository.

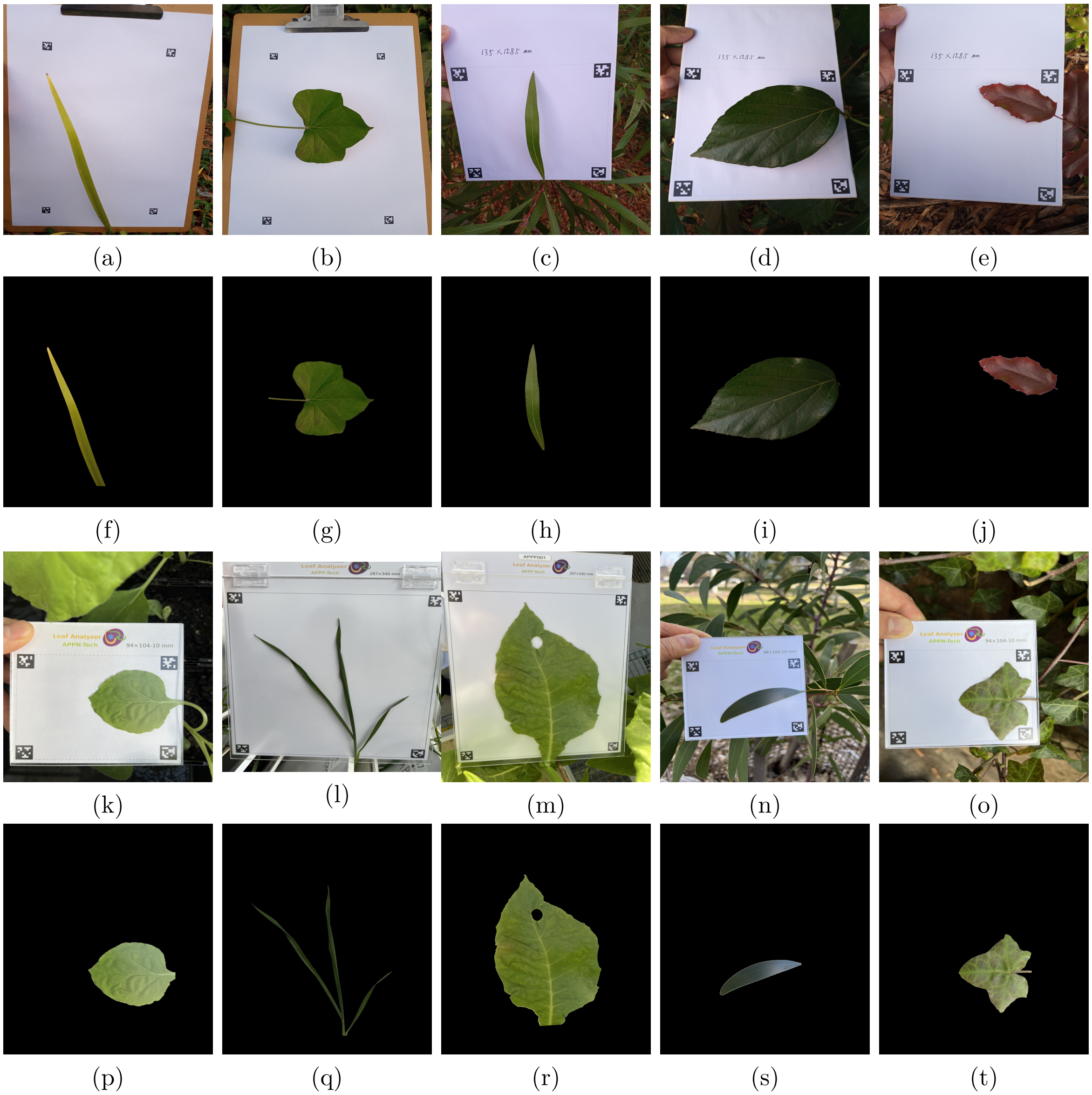

Figure S4: Non-destructive leaf area measurement in action. First row (a-e): original images captured with a pattern board. Second row(m-o): leaf segmentation results corresponding to first row images. Third row(k-o): original images taken with a pattern board (with plastic cover). Fourth row(p-t): leaf segmentation results corresponding to third row images.

Circle Index	Petiole Pro-Thresh	Petiole Pro-HSV	Leaf Analyzer
1	2080	2035	2016.5
2	2029	1991	2003.1
3	2005	1966	2004.5
4	2081	2044	2016.1
5	2017	1983	1983.7
6	1986	1957	1985.1
7	2072	2042	2001.5
8	2013	1983	1972.9
9	1998	1972	1988.6
10	2092	2059	2010.0
11	2029	2006	1983.6
12	2014	1993	1999.9

Table S1: Green circle area estimation results using Petiole Pro and Leaf Analyzer. All estimates are in mm2. The ground truth is 2000 mm2.

Image Index	LeafByte	Leaf Analyzer
1	40074.3	40077.5
2	39902.0	39917.2
3	40010.0	39973.7
4	40033.1	40019.8
5	39875.5	39988.8
6	40006.7	40011.0
7	40035.1	39981.1
8	40015.2	40067.6
9	39777.0	40120.9
10	39887.8	40062.7
11	39844.5	40004.1
12	39890.8	40011.2

Table S2: Green square area estimation results using LeafByte and Leaf Analyzer. All estimates are in mm2. The ground truth is 2000 mm2.

Video Demos

Video/Movie S1 Leaf morphological trait measurement demo.mp4 (Youtube link or Youku link)

Video/Movie S2 Leaf damage assessment demo.mp4 (Youtube link or Youku link)
